# Fortifying p53 – beyond Mdm2 inhibitors

**DOI:** 10.18632/aging.101073

**Published:** 2016-09-29

**Authors:** Anusha Sriraman, Yizhu Li, Matthias Dobbelstein

**Affiliations:** Institute of Molecular Oncology, Göttingen Center of Molecular Biosciences (GZMB), University Medical Center Göttingen, D-37077 Göttingen, Germany

**Keywords:** p53, Mdm2, PPM1D, Wip1, cancer treatment

The tumor suppressor p53 is mutated in roughly 50% of all human malignancies. However, in the other 50% of tumors which retain wildtype p53, it appears insufficiently active to confer tumor suppression, through cell cycle arrest or apoptosis. Much of this p53-inactivation occurs through the Mdm2 oncoprotein, the product of a p53-inducible gene. Mdm2 is an E3 ubiquitin-ligase that targets p53 for proteasomal degradation. In 2004, a small-molecule antagonist of Mdm2 was identified, known as Nutlin-3a or Nutlin. It binds to Mdm2 at the p53 binding pocket, thereby leading to activation of p53 and its target genes [2]. Recently, similar Mdm2 antagonists were taken to clinical trials, such as RG7388 (NCT02633059, NCT02407080, NCT02828930, NCT02670044, NCT02545283, NCT02624986), HDM201 (NCT02780128, NCT02143635), and MI-773 (NCT01636479), but the results regarding their efficacy have not been reported so far. Thus, delivering a wake-up call to dormant p53 in tumors remains a tempting but currently not proven option for cancer therapy.

While Nutlin readily induces cell cycle arrest, it was found ineffective in causing apoptosis in most tumor cells tested, even when p53 was wild type [3]. This raises the need to fortify the ability of Mdm2 antagonists to induce the pro-apoptotic functions of p53. In analogy to Mdm2, Wip1 (Wild-type p53 induced phosphatase, also known as PPM1D) is another p53-inducible antagonist to p53, often overexpressed in p53-wildtype cancer cells. Wip1 belongs to the PP2C family of Mg^2+^/Mn^2+^-dependent serine/threonine phosphatases and causes the dephosphorylation of p53 at Ser 15, thereby reducing p53 activity. It also dephosphorylates Mdm2, resulting in even more efficient p53 inhibition [4]. In 2014, an allosteric inhibitor of Wip1 known as GSK 2830371 was identified. It binds to the structural flap domain of Wip1 and reduces tumor cell growth in lymphoma xenograft models, the breast cancer cell line MCF-7, and neuroblastoma cells [5].

**Figure 1:**
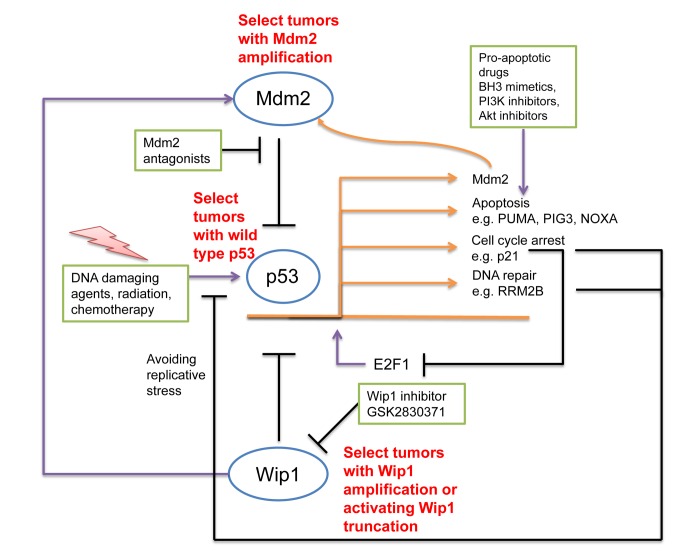
Strategies to fortify p53 in cancer therapy p53 activation occurs through most conventional chemo-therapeutics and irradiation, by DNA damage signaling. However, p53 activation is also achieved by inhibitors of the p53-antagonists Mdm2 and Wip1. p53, when active, promotes apoptosis or cell cycle arrest. On the other hand, a number of negative feedback loops attenuate p53. p53 activates the expression of Mdm2 and Wip1, and Wip1 further increases Mdm2 activity. Both Mdm2 and Wip1 antagonize p53. Moreover, p53 induces the CDK inhibitor p21, which impairs the activity of E2F1. Since E2F1 induces the Mdm2-antagonist p14/ARF and also some of the pro-apoptotic p53 target genes (e. g. NOXA), negative regulation of E2F1 attenuates some of p53's activities. Moreover, p21-induced cell cycle arrest prevents DNA replication and thus reduces DNA damage. Finally, p53 can promote DNA repair, consequently diminishing the efficacy of conventional chemotherapy. The fortification of p53 in this situation can be achieved by antagonists to Mdm2 and Wip1, but also through pro-apoptotic drugs. Such strategies are particularly promising in tumors that not only have wild type p53, but also amplifications of the Mdm2 gene and/or amplifications or activating truncations of Wip1.

In our study [1], we tested whether the simultaneous inhibition of both p53-antagonists, Mdm2 and Wip1, might induce p53 more potently than single inhibitors. And indeed, the combination of Nutlin and Wip1 inhibitor led to increased activity and stability of p53 that resulted in a major proportion of cells arresting at the G2/M phase of the cell cycle and/or undergoing senescence. Similar results were independently obtained by others [6, 7]. Thus, p53 activity can be fortified by the combined inhibition of factors that otherwise provide negative feedback on p53. This raises the perspective of interfering with p53-regulation at multiple levels (Fig. 1) to further boost p53 for cancer cell elimination.

The most traditional way of enhancing p53 activity in tumor cells consists in the initiation of a DNA damage response (DDR) by chemotherapy or irradiation. This activates DDR kinases – ATM, ATR, Chk1 and Chk2 – that target p53, resulting in p53 stabilization and activation. Future experiments might reveal whether genotoxic treatment will act synergistically when combined with inhibitors of Mdm2 and Wip1.

At present, even the combination of Nutlin and Wip1 inhibitor did not strongly induce apoptosis in the cells we analyzed. This setback may be caused, at least in part, by anti-apoptotic mechanisms frequently found in tumor cells. Future efforts might therefore include pro-apoptotic drugs such as BH3 mimetics or inhibitors of PI3 Kinase-Akt-signaling. Such strategies could complement p53 activation to induce cell death.

For successful application of Mdm2- or Wip1-inhibitors, the selection of responsive tumors might be essential. A wild type p53 status is an obvious requirement. Furthermore, tumors harboring amplified Wip1, or otherwise an activating truncation of Wip1, seem most promising regarding the successful use of a Wip1 inhibitor. These include breast cancer, neuro-blastoma, medulloblastoma, and melanoma. Further-more, Mdm2 antagonists appear most effective in tumors that contain amplifications of Mdm2, such as liposarcoma and osteosarcoma.

Of note, Nutlin can also confer protective effects on cells against chemotherapy. We and others have shown that Nutlin protects p53-proficient cells from the harmful effects of gemcitabine, taxanes, or Wee1 inhibition, at least in part by temporarily preventing the cells from entry into S phase or mitosis. The induction of DNA repair genes, such as Ribonucleotide reductase RRM2B, might further protect cells against genotoxic stress. Thus, care must be taken while combining Mdm2- and Wip1-inhibitors with conventional chemotherapeutics, e. g. by choosing a scheme where genotoxic drugs are applied before the inhibitors of Mdm2 or Wip1.

Enhancing p53 activity still appears like an attractive strategy to eliminate tumor cells that retain wild type p53 status. However, interfering with the Mdm2-p53-interaction often appears insufficient to eliminate tumors. Targeting additional antagonists of p53, in combination with genotoxic stress and pro-apoptotic strategies, might fortify p53 to the point where it induces cancer cell death.
